# Effects of Ramadan fasting during third trimester on Doppler indices, non-stress test of fetus and perinatal outcomes

**DOI:** 10.12669/pjms.41.5.11529

**Published:** 2025-05

**Authors:** Rabia Jamil, Saba Mughal, Sonam, Saba Sohail, Nazli Hossain

**Affiliations:** 1 Rabia Jamil, MBBS, FCPS. Senior Registrar, Department of Obs & Gynae Unit-I, Civil Hospital Karachi (CHK)/Dow Medical College (DMC), Karachi, Pakistan; 2 Saba Mughal Lecturer, School of Public Health, Dow University of Health Sciences, Karachi; 3 Sonam Postgraduate Trainee, Department of Obs & Gynae Unit-I, Civil Hospital Karachi (CHK)/Dow Medical College (DMC), Karachi, Pakistan; 4 Saba Sohail, MBBS, FCPS, Ph.D. Professor, Department of Radiology, Civil Hospital Karachi (CHK)/Dow Medical College (DMC), Karachi, Pakistan; 5 Nazli Hossain, MBBS, FCPS, MBE. Professor, Department of Obs & Gynae Unit-I, Civil Hospital Karachi (CHK)/Dow Medical College (DMC), Karachi, Pakistan

**Keywords:** Fasting, Ramadan, Fetal Doppler, Non-stress test, Third trimester

## Abstract

**Objective:**

To assess the Effects of Ramadan fasting during third trimester on Doppler indices, non-stress test and perinatal outcomes.

**Methods:**

This prospective, cross-sectional research was carried out in the Department of Obstetrics and Gynaecology unit 1, Dr Ruth KM Pfau Civil Hospital Karachi from March 2024 to May 2024. The study involved 100 participants they were divided into two groups, fasting group with minimum 10 days of fasting (Group-I) and non-fasting group (Group-II). Umbilical artery systolic/diastolic ratio, pulsatility index, middle cerebral artery pulsatility index and amniotic fluid index was measured during third trimester. This was followed by for non-stress test (cardiotocography) for about 30 minutes. Both groups were followed till delivery. At delivery placental weight, baby birth weight, head circumference, length and mid arm circumference of baby were recorded.

**Results:**

A total of 100 pregnant women were included. 52% (n=26) observed continuous fasting and 48% (n=24) observed intermittent fasting. Overall median age and BMI of pregnant women were 27 (IQR: 23-30) years and 25 (IQR: 24-29) kg/m^2^, respectively. Amniotic fluid index was a bit higher in women who were not fasting (Median: 13.7, IQR: 11.0-15.0) as compared to those who fasted during pregnancy (Median: 12.3, IQR: 10.7-15.0). Although Doppler assessment and CTG findings did not show any significant association with the fasting status of pregnant women except median pulsatility index which was significantly higher in non-fasted group (1.0, IQR: 0.9-1.4 vs. 0.9, IQR: 0.7-1.1, p-value=0.002). Overall median weight of baby and Apgar score at 5-min were 3.0 (IQR: 2.8-3.2) kg and 8 (IQR: 8-10), respectively. The anthropometric measurements were similar in both groups.

**Conclusion:**

Maternal fasting during Ramadan does not adversely affect Doppler indices, amniotic fluid index, non-stress test and fetal condition.

## INTRODUCTION

Ramadan is a month of fasting and it is one of the basic five fundamental pillars of Islam.^1^ During Ramadan all Muslims who fast, abstain from fluid, food, smoking and oral medications from sunrise to sunset. Islamic rules state that fasting is mandatory for adults with good health; however, patients, travelers, pregnant and lactating women are exempted from this rule. Pregnant women can make their own choice between fasting and non-fasting. They are worried about the effects of fasting on the fetus and pregnancy and possible complications. The extent to which fasting effects pregnancy and their fetal outcome is less known. As this is less investigated, health care provider also lack knowledge about the effects of Ramadan fasting. They fail to communicate or give advice to such patients.

Many pregnant women who choose to fast do not inform their health practitioners about their fasting state keeping in mind the fear of disagreement and rejection. Prolong periods of fasting without food intake in pregnancy is associated with increased in the level of maternal corticotrophin releasing hormone concentration which is linked with maternal and fetal health consequences. Release of increased maternal corticotrophin has been found to be associated with preterm labor. Maternal undernutrition during fasting especially in the second and third trimesters have also been found to affect the adult programming of different diseases.^2^ But there is lack of studies for evidence-based conclusion, and requires more extensive research to show the effect of Ramadan fasting on adult programming.^3^ Fasting results in maternal dehydration, and fluctuating glucose levels which may theoretically affect fetal growth. Past studies have shown markedly conflicting results regarding the effect of fasting on amniotic fluid volume,^3^,^4^ hyperemesis, five minutes Apgar score, fetal biometry parameters and birth weight,^5^ duration of gestation,^6^ and nonstress test.^7^ Hence making it a controversial aspect of obstetric healthcare with potential effect on fetal growth and perinatal outcome.

Obstetric management in conditions requiring fetal growth surveillance are reliably guided by Umbilical artery (UA) and fetal middle cerebral artery (MCA)flow Doppler studies.^8^ The umbilical artery diastolic flow progressively increases with advanced pregnancy causing a fall in the arterial resistance, and pulsatility to bring more blood to the rapidly growing fetus. This is measured in terms of pulsatility index (PI, ratio of peak umbilical systolic flow to minimum diastolic flow) and systolic to diastolic flow ratio(S/D) ratio representing the relative amount of blood during systole and diastole.^8^ Fetal MCA flow PI increases. These values have also been studied with in Muslim fasting women again with conflicting results.^9^,^10^

Similarly, the studies have been conducted to see the perinatal effects of maternal fasting. Savitri et al and Ziaee et al. did not find any significant difference in both groups of Ramadan fasting on birth weight, head circumference, length and five minutes apgar score.^11^ We have earlier reported a non-significant increase in preterm birth among women who fasted during pregnancy.^12^ Though in a larger cohort, gestational age was not found to be affected.^6^ A recent study from Germany found a decrease in birth weight of newborns, whose mothers fasted, specially during first trimester.^13^ (-352.92g, 95% CI: -537.38; -168.46).

The effects of fasting on birth weight during second and third trimester were not conclusive. The authors did not find any association of decreased birth weight with dietary modification during Ramadan. Savitri et al, found a non-significant decrease in birth weight of babies whose mother fasted during the second and third trimester.^11^ We have also found decreased placental weight among fasting mothers.^5^ This effect on birth weight extends into earlier part of childhood as well. In a multiple indicator cluster survey, the effect of fasting in later part of pregnancy was associated with decreased incidence of stunting, compared to fasting in earlier part of pregnancy.^14^ Few meta-analysis and scoping reviews conducted on fetal biometry and amniotic fluid indices, also did not find any conclusive evidence whether maternal fasting affects the above fetal parameters to provide evidence -based guidelines for health care workers.^15^ There is dearth of studies focusing on fetal growth, using Doppler indices, among pregnant fasting mothers.

As the expectant mothers seek advice on fasting from the health care providers, it is imperative to have an evidence-based knowledge backed by results in any indigenous population which should be exposed to same climatic and environmental situation rather than another geography and topography. To address this knowledge gap created by markedly varying results of fasting in pregnancy, the objective of this study was to see the effects of maternal fasting on fetal growth by measuring doppler indices, and perinatal outcome.

## METHODS

This prospective, cross-sectional research was carried out in the Department of Obstetrics and Gynaecology Unit-1, Dr Ruth KM Pfau Civil Hospital Karachi from March 2024 to May 2024. Participants were divided into two groups, fasting group with minimum 10 days of fasting (Group-I) and non-fasting group (Group-II). They were referred for the ultrasound Doppler of umbilical artery after 32 weeks, during day time. Following parameters were recorded, umbilical artery systolic/diastolic ratio, pulsatility index, middle cerebral artery pulsatility index and amniotic fluid index. This was followed by for non-stress test (cardiotocography) for about 30 minutes. Measurements like fetal basal heart rate, number of accelerations, decelerations and presence of contraction were recorded.

Women in both groups received iron and calcium supplements, and belonged to the same socio-economic groups. Both groups were followed till delivery. At delivery placental weight, baby birth weight, head circumference, length and mid arm circumference of baby were recorded. Women with low risk pregnancies and willing for delivery at the hospital were included. Women with medical conditions like diabetes mellitus, hypertension, thyroid disorders were excluded. Also women with multiple pregnancies were excluded.

### Ethical Approval:

The study was approved by the Institutional Review Board. (Ref: IRB 3447/DUHS/Approval/2024/82, dated March 21, 2024).

### Sampling technique:

Non probability consecutive sampling technique.

### Operational definition:

### SD ratio:

It is the measurement of the ratio of peak umbilical systolic velocity with the diastolic flow and identifies the amount of resistance in the placental vasculature. It was measured as the ratio of highest systolic velocity / diastolic velocity.^16^

### Pulsatility index: (Gosling index):

It is the difference between the peak systolic flow velocity (PSV) and minimum end diastolic flow velocity (EDV) measured as (PSV - EDV) / TAV.^16^

### Amniotic fluid index:

It is the sum of the four quadrant measurements (in cm) of pockets of maximal depth of amniotic fluid (free of an umbilical cord and fetal parts), in the gestational sac arbitrarily divided into four quadrants.

### Non stress test:

It is a test in pregnancy for about 20-30 minutes that measures fetal heart rate in response to movement and contractions. The result will be either reactive or non-reactive.

### Reactive NST:

Two or more accelerations with a heart rate of more than 15 beats, lasting at least 15 seconds within 20 minutes after 32 weeks of gestation, and without deceleration.

### Non-reactive NST:

Less than two accelerations and/or presence of decelerations.

### Low birth weight:

Birth weight of less than 2500gram.

### Preterm delivery:

Delivery before 37 weeks of gestation.

### Mid arm circumference:

It is the circumference of the right upper arm measured at the midpoint between the tip of the shoulder and tip of the elbow. The average is 13.5cm.

### Sample size:

We used open Epi calculator (version 3) for sample size calculation. By using mean and standard deviation for number of accelerations for fasting women was 6±3.2 and 4.08±3.3 for non-fasting group with 80% power and 95% confidence level and significance level(alpha) of 0.05 5%, a sample size of 92 women was calculated, the minimal sample size for each group was 46. However, a count of 100 samples with 50 individuals in each group was taken.^3^

### Data collection and Data analysis:

Data collection was started after taking an Ethical approval from Institutional Review Board. Written informed consent were taken from the participants. Data was collected by the principal investigator and co-investigator and all the findings were recorded in a self-design questionnaire. Both quantitative and qualitative variables such as maternal age, gestational age, parity, education status, umbilical artery SD ratio, pulsatility index, single deepest vertical pocket of amniotic fluid, fetal heart rate, number of accelerations, number of decelerations, number of contractions, preterm delivery, birth weight, Apgar score and admission to neonatal intensive care unit were included in questionnaire.

### Statistical analysis:

Data was analyzed using Statistical Package for Social Sciences version 27. Descriptive statistics were reported as percentage (frequency) for categorical variables, and median (IQR: inter-quartile range) for quantitative variables. Assumption of normality was checked for quantitative variables by Shapiro Wilk test. Mann-Whitney U test and Chi-square/ Fisher exact test were used to determine the relationship of the maternal/ neonatal outcomes and Doppler assessment with the fasting status of pregnant women during pregnancy. All test results having p-values ≤ 0.05 were considered statistically significant.

## RESULTS

A total of 100 pregnant women were included in this study. Half of them fasted in Ramadan during pregnancy, 52% (n=26) observed continuous fasting and 48% (n=24) observed intermittent fasting. Overall median age and BMI of pregnant women were 27 (IQR: 23-30) years and 25 (IQR: 24-29) kg/m^2^, respectively. Proportion of being educated as intermediate and above was slightly greater in non-fasted group (16%, n=8) as compared to fasted group (12%, n=6). Emergency LSCS was performed in 70% (n=35) pregnant women of non-fasted group whereas 58% (n=29) of fasted group (Table-I).

**Table-I:** Maternal characteristics by fasting and non-fasting group

Characteristics		Total	Fasting	Non-fasting	Test statistics (Z/χ^2^)	Effect size (r/V)	p-value*
		n	(n = 50)	(n = 50)			
Age (years)			27 (23 - 30)	28 (23 - 30)	-0.447	-0.045	0.655
BMI (kg/m^2^)			26.0 (24.4 - 29.5)	25.0 (23.8 - 28.3)	-1.045	-0.105	0.296
MUAC (cm)			27.5 (24.0 - 31.2)	29.0 (26.0 - 32.0)	-1.118	-0.112	0.263
Education							
	Unable to read & write	37	19 (38.0)	18 (36.0)	0.360	0.060	0.948
	Primary	28	14 (28.0)	14 (28.0)			
	Secondary	21	11 (22.0)	10 (20.0)			
	Intermediate & above	14	6 (12.0)	8 (16.0)			
Monthly income (PKR)							
	< 20000	28	17 (34.0)	11 (22.0)	1.891	0.135	0.413
	21000 - 50000	66	30 (60.0)	36 (72.0)			
	>50000	6	3 (6.0)	3 (6.0)			
Days of fasting			18.0 (13.7-29.0)	-			
Type of fasting							
	Continuous		26 (52.0)	-			
	Intermittent		24 (48.0)	-			
Gestational age (weeks)			38 (37 - 38)	38 (38 - 38)	-0.891	-0.089	0.373
Parity			1.5 (0 - 2.2)	2.0 (1.0 - 3.0)	-1.308	-0.131	0.191
Induction							
	Yes	3	2 (4.0)	1 (2.0)	0	0.059	0.999
	No	97	48 (96.0)	49 (98.0)			
Mode of delivery							
	SVD	25	15 (30.0)	10 (20.0)	1.653	0.129	0.437
	Elective LSCS	11	6 (12.0)	5 (10.0)			
	Emergency LSCS	64	29 (58.0)	35 (70.0)			

Data is given as median (Q_1_ - Q_3_) and n (%). BMI=Body Mass Index, MUAC=Mid-Upper Arm Circumference, SVD=Spontaneous Vaginal Delivery, LSCS=Lower Segment Cesarean Section. *p-value calculated by Mann-Whitney U test and Chi-square/ Fisher exact test.

Amniotic fluid index was a bit higher in women who were not fasting (Median: 13.7, IQR: 11.0-15.0) as compared to those who fasted during pregnancy (Median: 12.3, IQR: 10.7-15.0). Although Doppler assessment and CTG findings did not show any significant association with the fasting status of pregnant women except median pulsatility index which was significantly higher in non-fasted pregnant women group as compared to fasted group (1.0, IQR: 0.9-1.4 vs. 0.9, IQR: 0.7-1.1, p-value=0.002) (Table-II).

**Table-II:** Doppler assessment and CTG findings by fasting and non-fasting pregnant women group.

Variables	Total	Fasting	Non-fasting	p-value*
	n	(n = 50)	(n = 50)	
Doppler indices				
Umbilical artery S/D ratio		2.6 (2.3 - 3.2)	2.6 (2.4 - 2.9)	0.658
Pulsatility index		0.9 (0.7 - 1.1)	1.0 (0.9 - 1.4)	0.002
MCA pulsatility index		1.6 (1.0 - 2.3)	1.6 (1.1 - 2.0)	0.725
Amniotic fluid index		12.3 (10.7 - 15.0)	13.7 (11.0 - 15.0)	0.305
Cardiotocography				
Fetal basal heart rate		140 (130 - 140)	140 (133 - 140)	0.520
No. of accelerations		1 (1 - 2)	1 (1 - 2)	0.288
No. of decelerations		0 (0 - 0)	0 (0 - 0)	0.999
Presence of contraction		0 (0 - 0)	0 (0 - 0)	0.155
Repeat CTG				
Yes	17	7 (14.0)	10 (20.0)	0.424
No	83	43 (86.0)	40 (80.0)	

Data are given as median (Q1 - Q3) and n (%). CTG=Cardiotocography, MCA=Middle Cerebral Artery.

* p-value calculated by Mann-Whitney U test and Chi-square test.

Overall median weight of baby and Apgar score at 5-min were 3.0 (IQR: 2.8-3.2) kg and 8 (IQR: 8-10), respectively. Neonatal outcomes were compared between the groups of fasted and non-fasted pregnant women but none of the variable showed significant association with the fasting status of pregnant women (Table-III).

**Table-III:** Neonatal outcomes by fasting and non-fasting pregnant women group.

Variables	Total	Fasting	Non-fasting	p-value*
	n	(n = 50)	(n = 50)	
Weight of baby (kg)		2.9 (2.7 - 3.2)	3.0 (2.8 - 3.2)	0.576
Length of baby (cm)		41 (40 - 43)	41 (40 - 42)	0.986
Head circumference (cm)		32 (30 - 34)	32 (30 - 34)	0.630
MUAC (cm)		11 (10 - 12)	11 (10 - 12)	0.647
Weight of placenta (g)		700 (600 - 800)	700 (600 - 700)	0.350
Apgar score at 5 minutes		8 (8 - 10)	8 (8 - 9)	0.293
Gender of baby				
Male	53	30 (60.0)	23 (46.0)	0.161
Female	47	20 (40.0)	27 (54.0)	

Data are given as median (Q1 - Q3) and n (%). MUAC=Mid-Upper Arm Circumference.

* p-value calculated by Mann-Whitney U test and Chi-square test.

## DISCUSSION

This year Ramadan was observed in the months of March and April. The average duration of fast was more than 14 hours. The study included cohort of women in their late twenties with average gestational age of 37-38 weeks, mostly belonging to a lower socioeconomic status who were mostly either not or barely able to read and write. The BMI of the fasting group was slightly higher than the non-fasting group without reaching statistical significance.

Our study found that women with higher levels of education did not fast. This negative association with higher levels of education has also been reported in other studies.^17^ Similar association is also seen with socioeconomic status. Women with low socioeconomic status are found to fast more regularly, compared to women with high income groups.^7^ We found amniotic fluid index to be higher in control group of women. This has been observed in other studies as well. Sakar et al have also shown a significant difference in the amniotic fluid index between fasting and non-fasting mothers.^4^ The amniotic fluid depends on fetal urinary production. A decrease in maternal utero-placental perfusion due to hypovolemia may also lead to changes in the fetal renal blood flow. Researchers have shown that fetal renal artery’s blood flow is affected with Ramadan fasting.

The pulstaility index was found to be higher in fasting women, compared to the control group.^18^ The increase was significant in the second and fourth weeks of Ramadan. This may be a plausible reason for decrease in the amniotic fluid volume in fasting women, as it is associated with prolonged hours of dehydration and hypovolemia. In a much smaller group of women, Moradi did not find any change in the amniotic fluid volume between fasting and non-fasting women.^9^ In a larger cohort of women, decrease in the amniotic fluid resulting in oligohydramnios has been reported.^19^ The findings by Sakar et al, matches with our finding, where an increase in the amniotic fluid volume was observed in the non-fasting group of women.^4^

The relatively unaffected Doppler flow indices results might be explained by considering another study which evaluated the effect of meal intake in pregnancy and found diurnal variation in umbilical flow indices with no effect caused by meal intake.^20^ The only significant difference was found as higher PI in the fasting women. This may be related to the dehydration and hypovolemia caused by fasting in the hot summer months. Mirghani et al., also evaluated PI of uterine artery at an earlier gestational age between 20-24 weeks, among fasting and non-fasting, without any significant results.^21^ In another study of 240 women, divided in all three trimesters, no significant difference was found in umbilical artery systolic/ diastolic ratio on doppler measurements.^22^ This may be related to the dehydration and hypovolemia caused by fasting in the hot summer months. Among all the Doppler indices, PI is the one most affected by a number of factors including mothers’ age, BMI, parity, and smoking. In the present study, all the Doppler studies were carried out at around noon time, by reserved appointment, which minimized the diurnal variation.

Our study did not find much difference in the Non-Stress Test among both groups of women. Though the number of repeat NST was greater among fasting women, but it did not reach statistical significance. Decrease in the number of accelerations has been attributed to the decreased maternal and fetal glucose levels, and decreased fetal breathing movements.^23^ Repeat NST does increase the maternal stress level and workload on the medical staff members. The anthropometric were similar in both groups of women. This has also been reported by our group in an earlier study.^5^ In a prospective cohort from Netherlands, a decrease in birth weight was reported among fasting women, but it was not of statistical significance.^11^ The investigators concluded, that fasting during early months of pregnancy may decrease birth weight in newborns.

Our study highlights that doppler indices and perinatal outcome of pregnant women during Ramadan fasting do not show any significant changes, except for a subtle increase in pulsatility index, compared to non-fasting pregnant women. A larger multi center sample size, with diverse temperature is needed, to further strengthen the results.

### Strengths of study:

The main study strength is the prospective longitudinal data collection where every expectant mother was followed up till delivery, as well as the sizeable sample. This single center study included women who belonged to the similar socio-economic class, thus removing bias for nutritional status of the study group. The ultrasound operators were the same women imaging dedicated sonologists for all the studied women, removing the effect of inter-operator variation.

### Limitations:

The main limitation is that their smoking/ non-smoking exposure status was not determined which is known to effect the fetoplacental flow. We also did not take into account effect of temperature and heat stress as the study population included women in all three trimesters. The effect of heat stress on preterm labor and fetal growth are well established. Fetal biophysical profile score was also not determined which may be the subject of another research.

## CONCLUSION

Maternal fasting during Ramadan does not adversely affect Doppler indices, amniotic fluid index, non-stress test and fetal condition.

### Authors Contribution:

**NH:** Conceived the idea, manuscript writing and responsible for the accuracy and integrity of the study. **RJ:** Synopsis writing. Literature Search. **SM:** Performed statistical analysis of data. Critical Review. **SS:** Helped in sonology interpretation and final manuscript writing. **S:** Helped in data collection and follow up of participants. All authors have read and approved the final version.
